# An Efferocytosis-Associated Gene Signature for Identifying At-Risk MASH: Transcriptomic and Exploratory Plasma Biomarker Assessment

**DOI:** 10.3390/genes17080948

**Published:** 2026-08-13

**Authors:** Jingjing Jiang, Xianhua Mao, Weiqian Lou, Weiwei Lou, Ziqiang Li, Xinrong Zhang, Qing Xie, Rongtao Lai

**Affiliations:** 1 Department of Infectious Diseases, Ruijin Hospital, Shanghai Jiao Tong University School of Medicine, Shanghai 200025, China; jjingjiang@163.com (J.J.); louloulwq@126.com (W.L.); 13186581750@163.com (W.L.); aalii@126.com (Z.L.); 2 Division of Gastroenterology and Hepatology, Stanford University Medical Center, Palo Alto, CA 94304, USA; xhmao@stanford.edu; 3 Guangdong Provincial Key Laboratory of Food, Nutrition and Health, Department of Epidemiology, School of Public Health, Sun Yat-sen University, Guangzhou 510080, China; zhangxr259@mail.sysu.edu.cn

**Keywords:** at-risk metabolic dysfunction-associated steatohepatitis, efferocytosis, diagnostic biomarker, machine learning, non-invasive biomarkers

## Abstract

**Background/Objectives**: At-risk metabolic dysfunction-associated steatohepatitis (MASH) is associated with increased risks of cirrhosis, hepatocellular carcinoma, and liver-related mortality. Because impaired efferocytosis contributes to persistent hepatic inflammation and fibrotic remodeling in MASH, we investigated whether efferocytosis-associated molecular signatures could identify at-risk MASH. **Methods**: Bulk RNA-sequencing datasets from Gene Expression Omnibus (GSE135251 and GSE174478) were analyzed to identify differentially expressed efferocytosis-related genes and characterize associated pathways and immune infiltration patterns. Machine learning-based feature selection was used to identify hub genes, which were incorporated into a transcriptomic nomogram. Experimental validation involved reverse transcription-quantitative polymerase chain reaction (RT-qPCR) and Western blotting of liver tissues from a Western diet-induced murine metabolic dysfunction-associated steatotic liver disease (MASLD) model. Plasma proteomic data were analyzed to explore the discriminatory performance of hub gene products. **Results**: A total of 17 efferocytosis-related genes (ERGs) associated with at-risk MASH were identified and enriched in pathways related to efferocytosis, inflammation, and immune regulation. Five hub genes, *CD24*, *CHI3L1*, *TREM2*, *PTGS2*, and *LGR6*, were shared by all three feature-selection approaches and significantly upregulated in at-risk MASH. A transcriptomic nomogram yielded area under the curve (AUC) values of 0.866 and 0.805 in the training and external cohorts, respectively. In the murine MASLD model, all five hub genes showed increased mRNA and protein expression in advanced disease. Exploratory plasma proteomic analysis identified elevated circulating TREM2 and CHI3L1 levels in at-risk MASH, and a simplified plasma-based model achieved an AUC of 0.736. **Conclusions**: This study identified a five-gene efferocytosis-associated signature and developed models for identifying at-risk MASH, suggesting that these genes warrant further evaluation as candidate biomarkers.

## 1. Introduction

Metabolic dysfunction-associated steatotic liver disease (MASLD) has emerged as the most common chronic liver disease worldwide and is increasingly recognized as a major contributor to liver-related morbidity and mortality [1]. Within the MASLD spectrum, metabolic dysfunction-associated steatohepatitis (MASH) represents a progressive inflammatory phenotype characterized by hepatocellular injury, immune activation, and fibrosis of variable severity. Importantly, disease progression in MASH is highly heterogeneous, and not all individuals with steatosis develop clinically significant liver injury [2]. Patients with at-risk MASH, commonly defined by a nonalcoholic fatty liver disease activity score (NAS) ≥ 4 together with significant fibrosis (F ≥ 2), exhibit substantially increased risks of advanced fibrosis, cirrhosis, hepatocellular carcinoma, hepatic decompensation, and liver-related mortality [3,4]. Because fibrosis stage remains one of the strongest predictors of long-term clinical outcomes, early identification of patients with at-risk MASH is essential for timely intervention, clinical evaluation, and therapeutic decision-making.

Currently, liver biopsy remains the reference method for diagnosing and staging at-risk MASH. However, its clinical application is limited by several important drawbacks, including its invasive nature, sampling variability, interobserver heterogeneity, procedure-related risks, and poor feasibility for large-scale population screening or longitudinal disease monitoring [5,6]. Consequently, considerable efforts have been directed towards the development of non-invasive tests (NITs), including imaging-based modalities and plasma-derived scoring systems. Although widely used, existing NITs show variable diagnostic performance, particularly in distinguishing progressive steatohepatitis from less severe disease stages, and many primarily reflect indirect features such as metabolic dysfunction, hepatocellular injury, or fibrosis burden [7]. Importantly, most currently available biomarkers provide limited mechanistic insight into the underlying biological processes driving disease progression. Therefore, there remains a critical need to identify biologically informative molecular signatures that not only improve the identification of at-risk MASH but also enhance understanding of the pathogenic mechanisms underlying MASH progression [8].

Increasing evidence suggests that dysregulated immune responses and defective resolution of inflammation are central to the pathogenesis of at-risk MASH [9]. Efferocytosis refers to the phagocytic recognition and removal of apoptotic cells and is essential for hepatic homeostasis and the resolution of inflammation [10,11]. Efficient efferocytosis prevents secondary necrosis, limits the release of pro-inflammatory intracellular contents, and promotes pro-resolving macrophage reprogramming, thereby restricting chronic inflammation and fibrotic remodeling [12,13]. In the liver, macrophage-mediated clearance of apoptotic hepatocytes is particularly important in the setting of lipotoxic injury. Emerging studies have demonstrated that, during MASH progression, the rate of hepatocyte apoptosis may exceed the clearance capacity of macrophages, resulting in defective efferocytosis, accumulation of apoptotic debris, sustained immune activation, and progressive fibrogenesis [14,15]. Moreover, impaired efferocytosis has been associated with alterations in macrophage phenotypes, lipid handling, and inflammatory signaling pathways that contribute to the transition from simple steatosis to advanced steatohepatitis and fibrosis [14,15,16]. These findings provide a biological rationale for investigating genes involved in efferocytosis. However, whether such a biologically informed molecular signature can assist in identifying patients with at-risk MASH status remains insufficiently explored.

To address this gap, we applied an integrative multi-level strategy combining bioinformatic analyses, murine expression validation, and exploratory biomarker development. We identified efferocytosis-associated hub genes from public transcriptomic datasets, validated their expression in a Western diet-induced murine model of metabolic dysfunction-associated steatotic liver disease (MASLD), and further explored whether selected tissue-derived biomarkers could be incorporated into a plasma-based classification model. In addition, we investigated the immune and cellular expression characteristics of these candidate genes using immune infiltration analysis and single-cell RNA-sequencing datasets. Our findings suggest the potential relevance of efferocytosis-associated molecular signatures for identifying at-risk MASH, propose candidate biomarkers, and provide preliminary insights into potential mechanisms for future clinical and functional validation studies.

## 2. Materials and Methods

### 2.1. Data Sources

To identify efferocytosis-related genes (ERGs) associated with MASH, bulk RNA-sequencing datasets related to MASLD (GSE135251 and GSE174478) and single-cell RNA-sequencing (scRNA-seq) datasets derived from human liver tissue (GSE186328 and GSE136103) were retrieved from the Gene Expression Omnibus (GEO) database, a publicly accessible repository for high-throughput gene expression data (http://www.ncbi.nlm.nih.gov/geo; accessed on 11 June 2026). GSE135251 and GSE174478 were analyzed separately as the training and external validation cohorts, respectively. After excluding healthy controls, GSE135251 included 206 liver samples, comprising 110 samples with at-risk MASH and 96 not-at-risk samples. GSE174478 consisted exclusively of MASLD liver samples, and all 94 samples, including 50 at-risk and 44 not-at-risk samples, were included. Plasma proteomic data for exploratory plasma-based biomarker assessment were obtained from a previously published cohort of 191 patients, including 79 patients with at-risk MASH and 112 not-at-risk individuals [17]. Detailed dataset characteristics are summarized in Table S1.

ERGs were collected from GeneCards using “efferocytosis” as the search term and from Kyoto Encyclopedia of Genes and Genomes (KEGG) based on efferocytosis-related pathway annotations. Specifically, 147 and 157 ERGs were retrieved from GeneCards and KEGG, respectively. After duplicate removal, 242 unique ERGs were retained (Table S2).

### 2.2. Outcome Definition

The primary outcome was at-risk MASH within the MASLD population, defined histologically by NAS ≥ 4 and fibrosis stage F ≥ 2 [3]. Patients who did not meet these criteria were classified as not-at-risk individuals.

### 2.3. Differential Expression Analysis and Identification of Risk-Associated ERGs

Raw count data from GSE135251 were first scaled with the trimmed mean of M-values method implemented in the “edgeR” package (version 4.4.2) [18]. The normalized counts were then analyzed with the limma-voom framework implemented in the “limma” package (version 3.62.2) to estimate group-wise expression differences [19]. Differentially expressed genes (DEGs) were retained when the Benjamini–Hochberg-adjusted false discovery rate (FDR) < 0.05 and absolute log2 fold change (log2FC) > 0.5. Expression profiles were visualized as volcano plots and heatmaps using the “ggplot2” (version 4.0.0) and “pheatmap” (version 1.0.13) packages. Risk-associated ERGs were obtained by intersecting the list of DEGs with the predefined catalog of ERGs and retaining genes that differed between at-risk and not-at-risk MASH.

### 2.4. Functional Enrichment Analyses

To evaluate the sample-level activity of risk-associated ERGs, single-sample gene set enrichment analysis (ssGSEA) was performed using the “GSVA” package (version 2.2.1) in the GSE135251 dataset. Biological interpretation of these genes was performed through Gene Ontology (GO) and KEGG enrichment analyses using the “clusterProfiler” package (version 4.16.0). For GO and KEGG enrichment analyses, *p*-values were adjusted using the Benjamini–Hochberg procedure. For each hub gene, KEGG-based gene set enrichment analysis (GSEA) was performed by ranking genes according to their correlation with hub gene expression to explore pathway-level functional relevance.

### 2.5. Machine Learning for Hub Gene Identification

To identify candidate hub genes associated with at-risk MASH, three complementary feature selection approaches—stepwise regression, least absolute shrinkage and selection operator (LASSO) regression, and support vector machine-recursive feature elimination (SVM-RFE)—were applied using the “stats” (version 4.5.1), “glmnet” (version 4.1-10), “e1071” (version 1.7-16), and “caret” (version 7.0-1) packages. These methods were selected because they represent complementary feature-selection principles: likelihood-based stepwise regression provides a parsimonious and interpretable model, LASSO performs regularization and sparse variable selection, and SVM-RFE ranks features according to their joint contribution to classification performance. Bidirectional stepwise logistic regression was used to identify genes associated with at-risk MASH status, and odds ratios (ORs) with 95% confidence intervals (CIs) were calculated. LASSO regression was performed for feature selection, with the optimal penalty parameter selected at λ.min based on ten-fold cross-validation. For SVM-RFE, feature subsets were evaluated using five-fold cross-validation, and the subset with the highest cross-validated accuracy was selected. Candidate genes identified by each method were subsequently compared, and genes shared by all three approaches were defined as hub genes to reduce dependence on any single algorithm and improve cross-method selection consistency.

### 2.6. Nomogram Model Construction and Validation

Nomogram models were constructed using both transcriptomic and plasma proteomic datasets with the “rms” package (version 8.1-0). Receiver operating characteristic (ROC) curves were generated using the “pROC” package (version 1.19.0.1) to evaluate discriminatory performance. Individual total scores were calculated using the “nomogramFormula” package (version 1.2.0.0). Model calibration and potential clinical utility were assessed using calibration curves (“rms” package; version 8.1-0) and decision curve analysis (DCA; “rmda” package; version 1.6), respectively. The transcriptomic nomogram was developed in GSE135251, with internal calibration assessed using 1000 bootstrap resamples to obtain a bias-corrected calibration curve, and was externally validated in the independent GSE174478 cohort. The plasma-based nomogram was generated using a previously published proteomics dataset [17].

### 2.7. Animal Experiments and Validation

Eight-week-old male C57BL/6 mice were obtained from GemPharmatech Co., Ltd. (Nanjing, China). Animals were housed under specific-pathogen-free conditions with ad libitum access to food and water. Following acclimatization, mice were fed a Western diet containing 60% of kcal from fat for either 12 or 24 weeks to establish milder and more advanced diet-induced MASLD phenotypes, respectively, and to investigate hub gene expression changes during disease progression [15,20]. Liver tissues were subsequently collected for downstream analyses. All animal experiments were approved by the Ethics Committee of Ruijin Hospital, Shanghai Jiao Tong University School of Medicine (RJ20260116), and were conducted in accordance with institutional guidelines for the care and use of laboratory animals.

Liver histopathology was assessed using hematoxylin and eosin (H&E) staining and Masson’s trichrome staining. NAS was determined from H&E-stained sections based on steatosis, lobular inflammation, and hepatocyte ballooning. The Masson-positive collagen area was quantified using ImageJ software (version 1.53q).

Total RNA was extracted from liver tissue and analyzed using reverse transcription-quantitative polymerase chain reaction (RT-qPCR), with gene expression normalized to 18S rRNA using the 2^−ΔΔCt^ method. Primer sequences are listed in Table S3.

Protein expression was evaluated by Western blotting. Target proteins and the internal control β-tubulin were detected on the same membrane. Multiplex immunohistochemistry (mIHC) using a tyramide signal amplification-based detection system was performed to evaluate CD68 and triggering receptor expressed on myeloid cells 2 (TREM2) expression. Detailed antibody information is provided in Table S4.

### 2.8. Immune Infiltration and Single-Cell Analysis

Relative fractions of 22 immune-cell populations in GSE135251 were inferred using the Cell-type Identification by Estimating Relative Subsets of RNA Transcripts (CIBERSORT) algorithm with the LM22 reference matrix. Differences in inferred immune cell proportions between the at-risk and not-at-risk groups were assessed using the Wilcoxon rank-sum test. Relationships between hub gene expression and inferred immune fractions were assessed using Spearman’s rank correlation and displayed as a correlation heatmap.

scRNA-seq datasets (GSE186328 and GSE136103) were analyzed using the “Seurat” package (version 5.3.0). Cells were retained if they contained 300–10,000 detected genes, 500–10,000 unique molecular identifiers, <10% mitochondrial gene content, and <1% hemoglobin gene content. After quality control, datasets were normalized and integrated, followed by dimensionality reduction, clustering, and cell-type annotation based on canonical marker genes. Cell-type-specific expression patterns of hub genes were subsequently evaluated and visualized.

### 2.9. Molecular Regulatory Network Construction and Exploratory Drug–Gene Interaction Analysis

Transcription factors (TFs) targeting hub genes were predicted using the JASPAR database. miRNA–target interactions (microT score > 0.3) were retrieved from TarBase v9.0. TF-hub gene and miRNA–hub gene interaction networks were constructed and visualized using Cytoscape 3.10.3. Candidate drug–gene interactions involving the hub genes were retrieved from the Drug Gene Interaction Database (DGIdb).

### 2.10. Statistical Analysis

**Figure 1:**
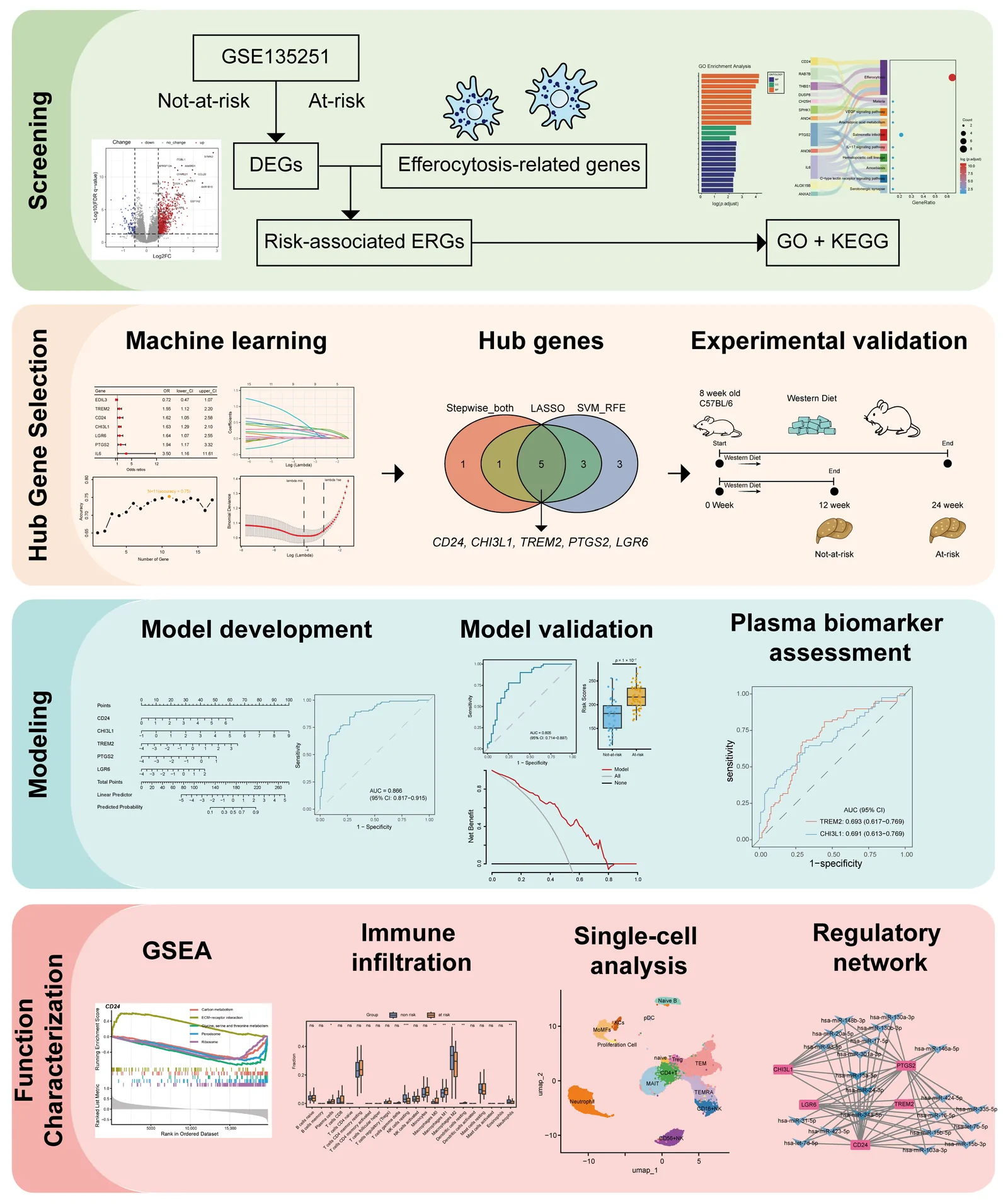
Study workflow. Schematic overview of the study design, including identification of efferocytosis-related genes (ERGs), differential expression and enrichment analyses, machine learning-based hub gene selection, construction and validation of a nomogram, murine validation experiments, immune infiltration analysis, single-cell RNA-sequencing analysis, and exploratory molecular regulatory network analysis. ns, not significant, * *p* < 0.05; ** *p* < 0.01; *** *p* < 0.001.

All statistical analyses were performed using R software (version 4.5.1) or GraphPad Prism (version 10.1.2). Normality and homogeneity of variance were assessed before parametric testing. Continuous variables satisfying these assumptions were compared using Student’s *t*-test; otherwise, the Wilcoxon rank-sum test was used. NAS scores were compared using the Mann–Whitney U test. Correlations were assessed using Spearman’s rank correlation coefficients. Statistical significance was defined as a two-sided *p* < 0.05. The overall experimental workflow is illustrated in Figure 1.

## 3. Results

### 3.1. Identification and Enrichment Analysis of Risk-Associated ERGs

**Figure 2:**
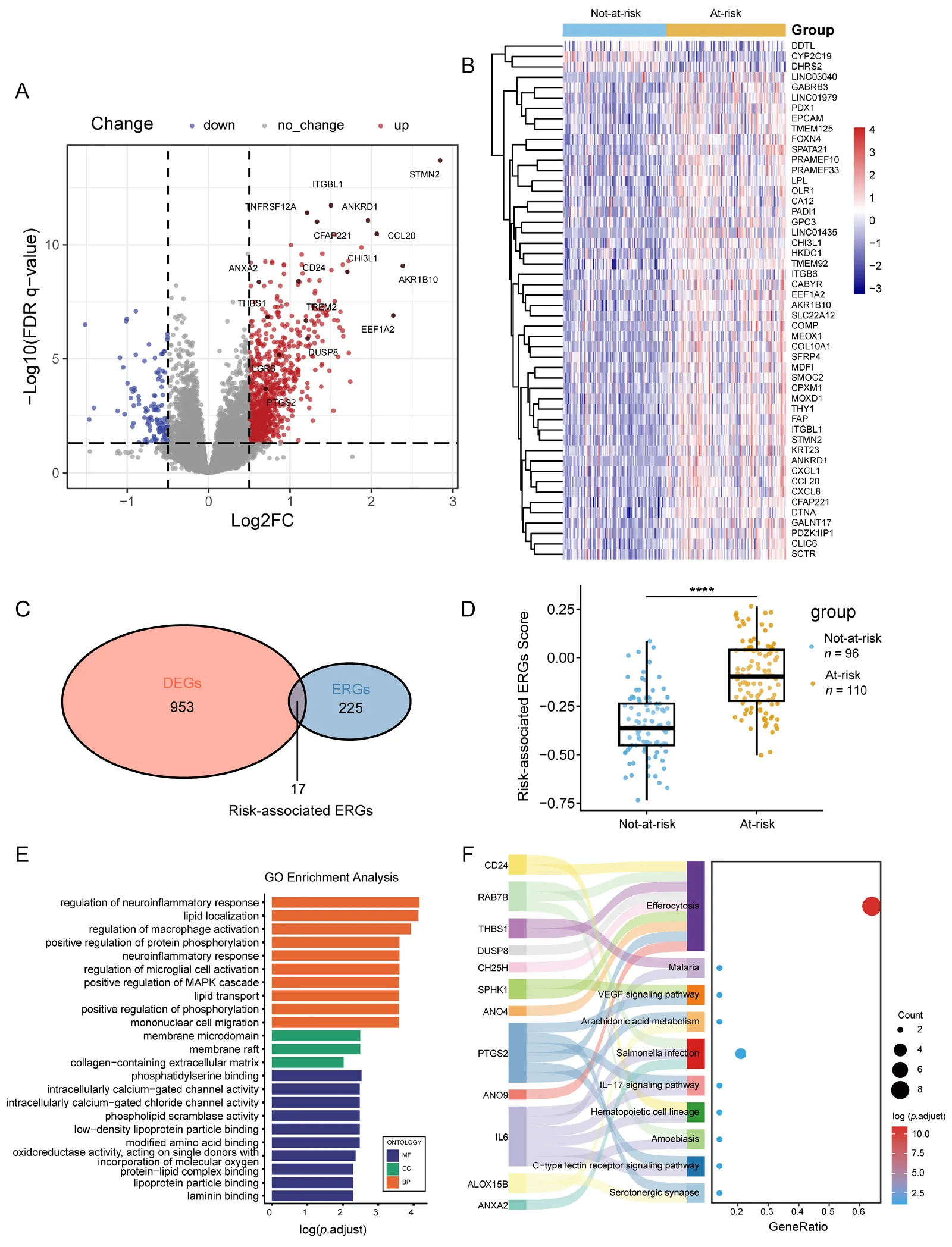
Identification and enrichment analysis of risk-associated ERGs: (**A**) Volcano plot showing the distribution of differentially expressed genes (DEGs) in the GSE135251 dataset. (**B**) Heatmap showing the expression patterns of the top 50 DEGs ranked by absolute log2 fold change (log2FC). (**C**) Venn diagram showing the overlap between DEGs and efferocytosis-related genes (ERGs). (**D**) Boxplot comparing single-sample gene set enrichment analysis (ssGSEA) scores of risk-associated ERGs between the not-at-risk and at-risk MASH groups. (**E**) Gene Ontology (GO) enrichment analysis and (**F**) Kyoto Encyclopedia of Genes and Genomes (KEGG) pathway enrichment analysis of risk-associated ERGs. Student’s *t*-test was used for group comparisons. Data are presented as mean ± standard deviation (SD). **** *p* < 0.0001.

Differential expression analysis of the GSE135251 dataset identified 970 DEGs, including 854 upregulated and 116 downregulated genes. The distribution of DEGs is illustrated in Figure 2A and Table S5. The heatmap in Figure 2B illustrates the expression patterns of the top 50 DEGs ranked according to absolute log2FC values. Intersecting DEGs with predefined ERGs identified 17 risk-associated ERGs (Figure 2C and Table S6).

ssGSEA demonstrated significantly higher enrichment scores for these ERGs in the at-risk MASH group than in the not-at-risk group (*p* < 0.001; Figure 2D). GO and KEGG enrichment analyses further showed that these ERGs were predominantly enriched in pathways associated with efferocytosis, inflammation, and immune regulation, including macrophage activation and interleukin (IL)-17 signaling (Figure 2E,F). These pathways are closely implicated in the progression from simple steatosis to steatohepatitis and fibrotic remodeling.

### 3.2. Machine Learning-Based Identification of Hub Genes

**Figure 3:**
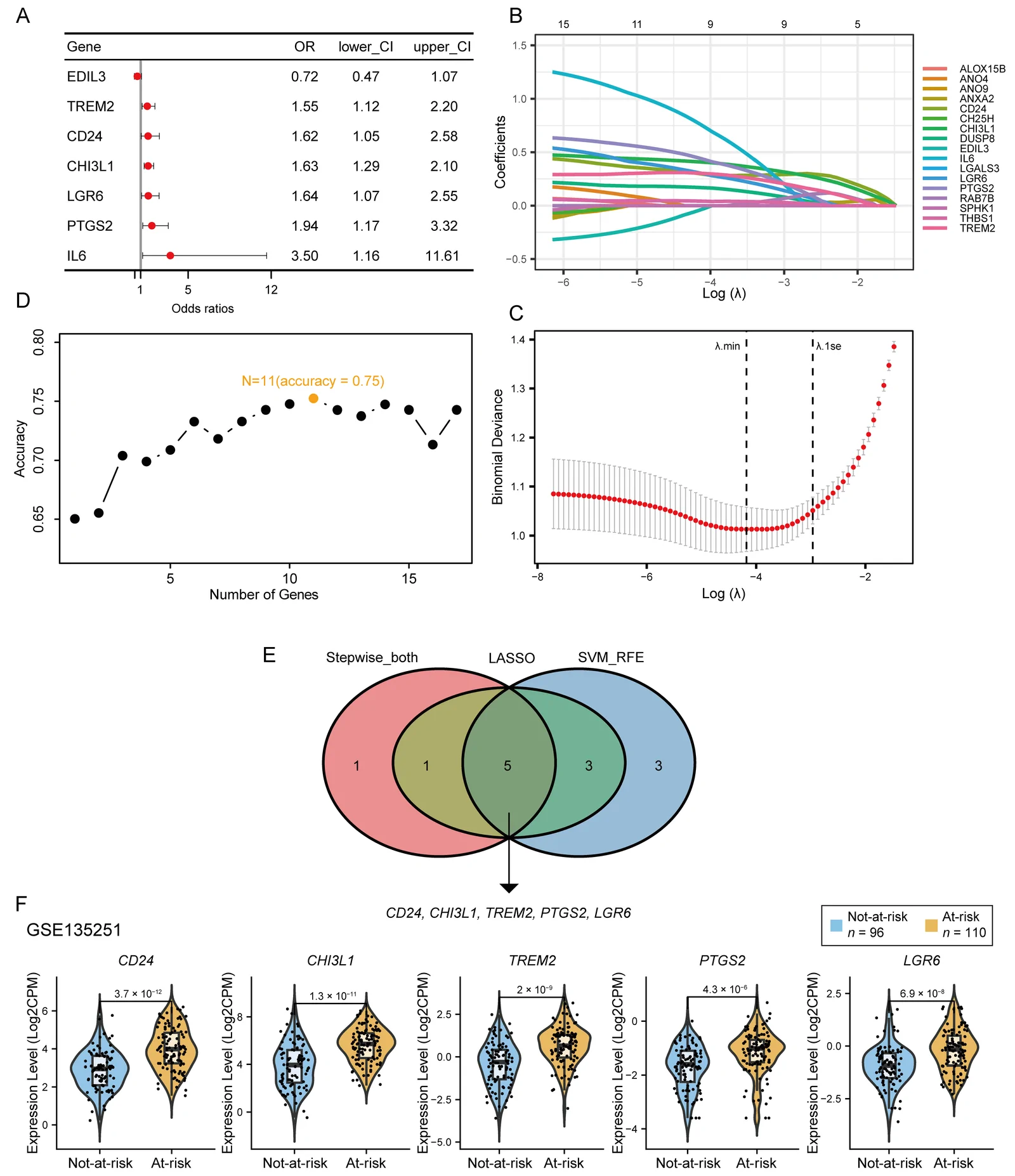
Machine learning-based identification of hub genes: (**A**) Forest plot showing odds ratios (ORs) and 95% confidence intervals (CIs) for genes identified by stepwise logistic regression analysis. (**B**) Regression coefficient profiles of predictor variables under different penalty parameters (λ) in the least absolute shrinkage and selection operator (LASSO) regression model. (**C**) Cross-validation plot used to determine the optimal λ value in the LASSO model. (**D**) Accuracy plot generated by support vector machine-recursive feature elimination (SVM-RFE) analysis. (**E**) Venn diagram showing the overlap of candidate genes identified by stepwise regression, LASSO, and SVM-RFE analyses. (**F**) Violin plots showing expression levels of the identified hub genes in the GSE135251 dataset. Student’s *t*-test was used for group comparisons. Data are presented as mean ± SD.

To further identify key genes associated with at-risk MASH, stepwise regression, LASSO regression, and SVM-RFE were applied to the 17 risk-associated ERGs in GSE135251. Stepwise logistic regression identified several candidate genes associated with at-risk MASH status (Figure 3A). LASSO regression analysis was subsequently performed, and the optimal penalty parameter was determined by cross-validation (Figure 3B,C). SVM-RFE analysis was then used to identify the optimal gene subset based on classification accuracy (Figure 3D).

Five genes, *CD24*, chitinase-3-like protein 1 (*CHI3L1*), *TREM2*, prostaglandin-endoperoxide synthase 2 (*PTGS2*), and leucine-rich repeat-containing G-protein-coupled receptor 6 (*LGR6*), were shared by all three feature-selection approaches (Figure 3E). All five genes were significantly upregulated in the at-risk MASH group (all *p* < 0.0001; Figure 3F).

### 3.3. Validation of Hub Genes in the MASLD Mouse Model

**Figure 4:**
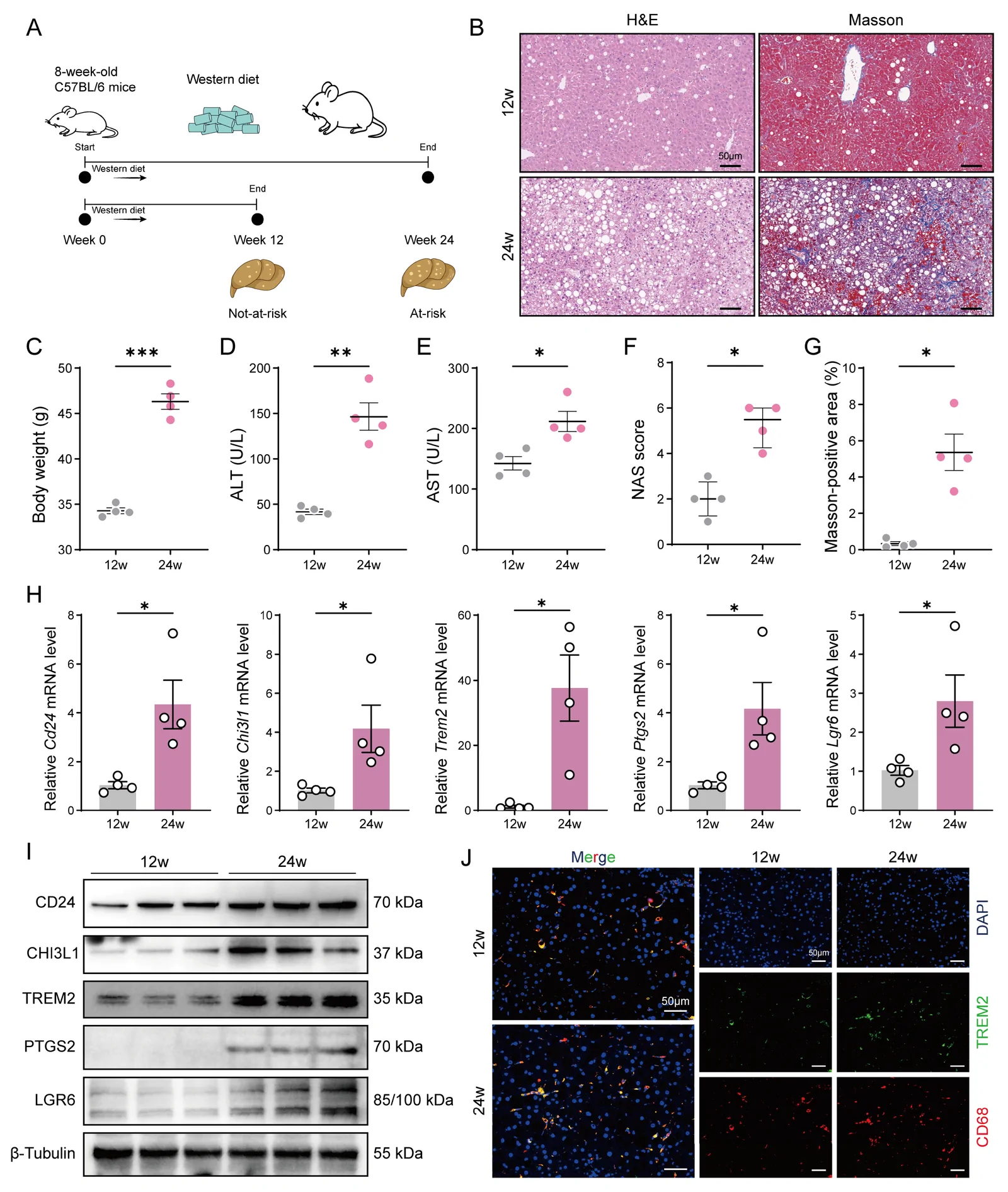
Validation of hub genes in the Western diet-induced MASLD mouse model: (**A**) Experimental design of the Western diet-induced metabolic dysfunction-associated steatotic liver disease (MASLD) mouse model. (**B**) Representative hematoxylin and eosin (H&E) staining and Masson’s trichrome staining of liver tissues from each group (scale bar = 50 μm). (**C**–**E**) Body weight and serum alanine aminotransferase (ALT) and aspartate aminotransferase (AST) levels in C57BL/6 mice fed a Western diet for 12 or 24 weeks (*n* = 4 per group). (**F**) Nonalcoholic fatty liver disease activity score (NAS) based on H&E staining, including steatosis, lobular inflammation, and hepatocyte ballooning. (**G**) Quantification of Masson-positive collagen area, expressed as the percentage of collagen-positive area relative to total tissue area. (**H**) Reverse transcription-quantitative polymerase chain reaction (RT-qPCR) analysis of relative mRNA expression levels of hub genes in liver tissues (*n* = 4 per group). (**I**) Western blot analysis of hub gene protein expression (*n* = 3 per group). (**J**) Representative multiplex immunohistochemistry (mIHC) images showing co-localization of TREM2 (green) and CD68 (red), with nuclei counterstained with DAPI (blue). Scale bar = 50 μm. Data are presented as mean ± standard error of the mean (SEM), except for NAS scores, which are presented as medians with interquartile ranges (IQR). Statistical significance was determined using an unpaired Student’s *t*-test for panels (**C**–**E**,**G**,**H**), and the Mann–Whitney U test for panel (**F**). * *p* < 0.05; ** *p* < 0.01; *** *p* < 0.001.

A Western diet-induced murine model of MASLD was established to evaluate changes in hub gene expression during disease progression in vivo (Figure 4A). Compared with mice fed a Western diet for 12 weeks, mice fed the diet for 24 weeks exhibited a more advanced steatohepatitis-like phenotype, characterized by increased hepatic steatosis, inflammatory activity, and collagen deposition on H&E and Masson’s trichrome staining (Figure 4B). Consistently, the 24-week group displayed significantly higher body weight, serum alanine aminotransferase (ALT) and aspartate aminotransferase (AST) levels, NAS scores, and Masson-positive collagen area than the 12-week group (Figure 4C–G).

RT-qPCR analysis confirmed significant upregulation of all five hub genes, namely *Cd24*, *Chi3l1*, *Trem2*, *Ptgs2*, and *Lgr6*, in the 24-week group (all *p* < 0.05; Figure 4H). Western blot analysis further demonstrated increased abundance of the corresponding proteins (Figure 4I). Given the close association of TREM2 with macrophage-mediated efferocytosis, TREM2 was selected for further cellular localization analysis [15]. mIHC showed co-localization of TREM2 with CD68^+^ hepatic macrophages, and the co-localization signal was stronger in the 24-week group (Figure 4J).

### 3.4. Construction and Evaluation of the Classification Model in GSE135251

**Figure 5:**
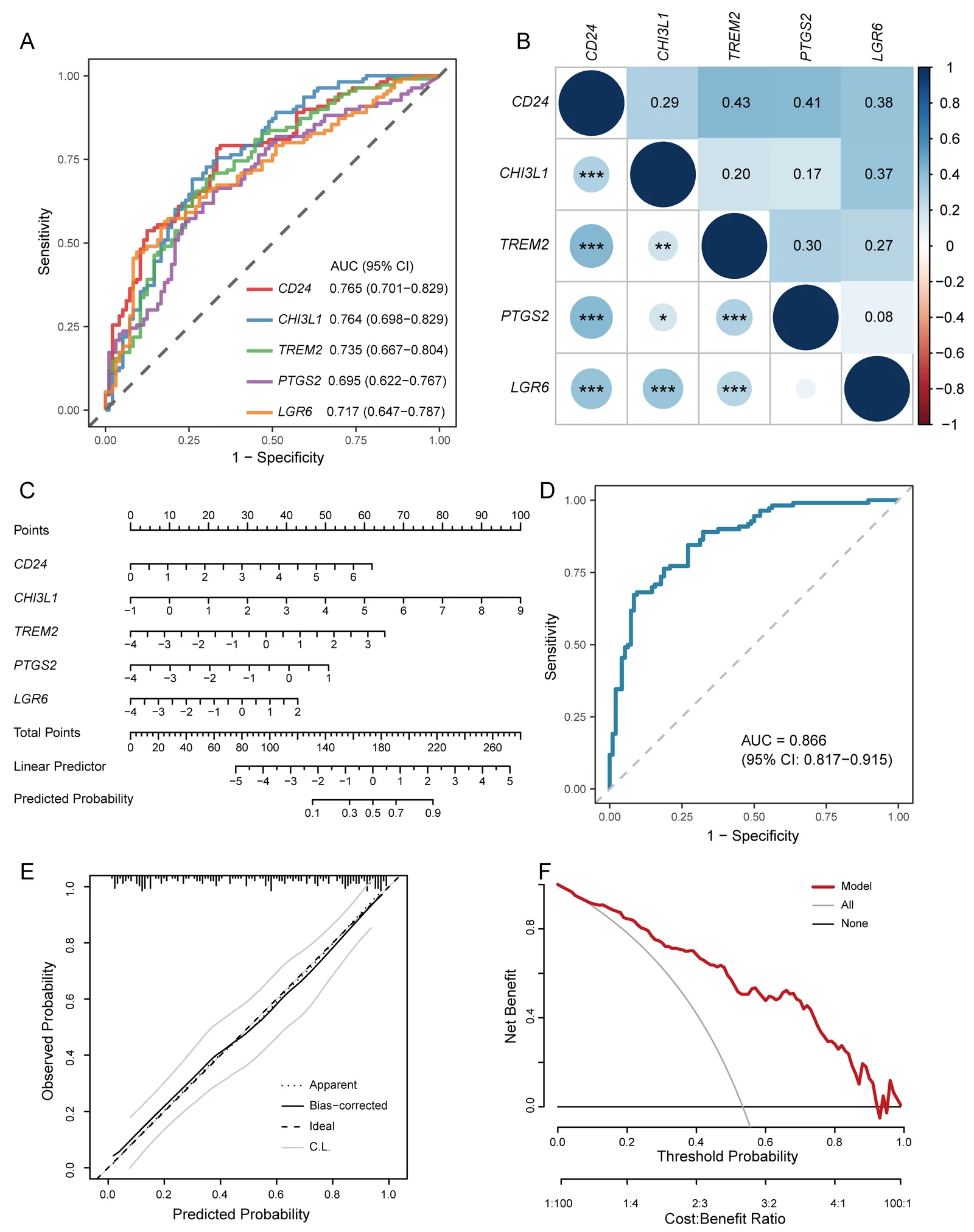
Construction and evaluation of the classification model for identifying at-risk MASH in GSE135251: (**A**) Receiver operating characteristic (ROC) curves of individual hub genes in the GSE135251 dataset. (**B**) Correlation heatmap showing pairwise correlations among hub genes. (**C**) Nomogram for calculating a risk score for at-risk MASH. (**D**) ROC curve evaluating the discriminatory performance of the nomogram model; the solid line represents the ROC curve, and the diagonal dashed line represents chance-level discrimination. (**E**) Calibration curve assessing agreement between predicted probabilities and observed outcomes. (**F**) Decision curve analysis (DCA) showing the potential clinical net benefit of the nomogram across different threshold probabilities. Correlation coefficients were calculated using Spearman’s rank correlation analysis. * *p* < 0.05; ** *p* < 0.01; *** *p* < 0.001.

The individual hub genes *CD24, CHI3L1, TREM2, PTGS2*, and *LGR6* showed moderate discriminatory performance for identifying at-risk MASH, with area under the curve (AUC) values ranging from 0.695 to 0.765 (Figure 5A). Pairwise correlation analysis demonstrated low correlations among the hub genes, supporting their incorporation into a combined classification model without substantial collinearity (all pairwise correlation coefficients < 0.6; Figure 5B).

All five hub genes were subsequently integrated into a nomogram model (Figure 5C), which achieved an AUC of 0.866 (95% CI: 0.817–0.915) in the training cohort (Figure 5D). The model also demonstrated good calibration (Figure 5E) and provided clinical net benefit across a range of threshold probabilities according to DCA (Figure 5F).

### 3.5. External Validation of the Nomogram in GSE174478

**Figure 6:**
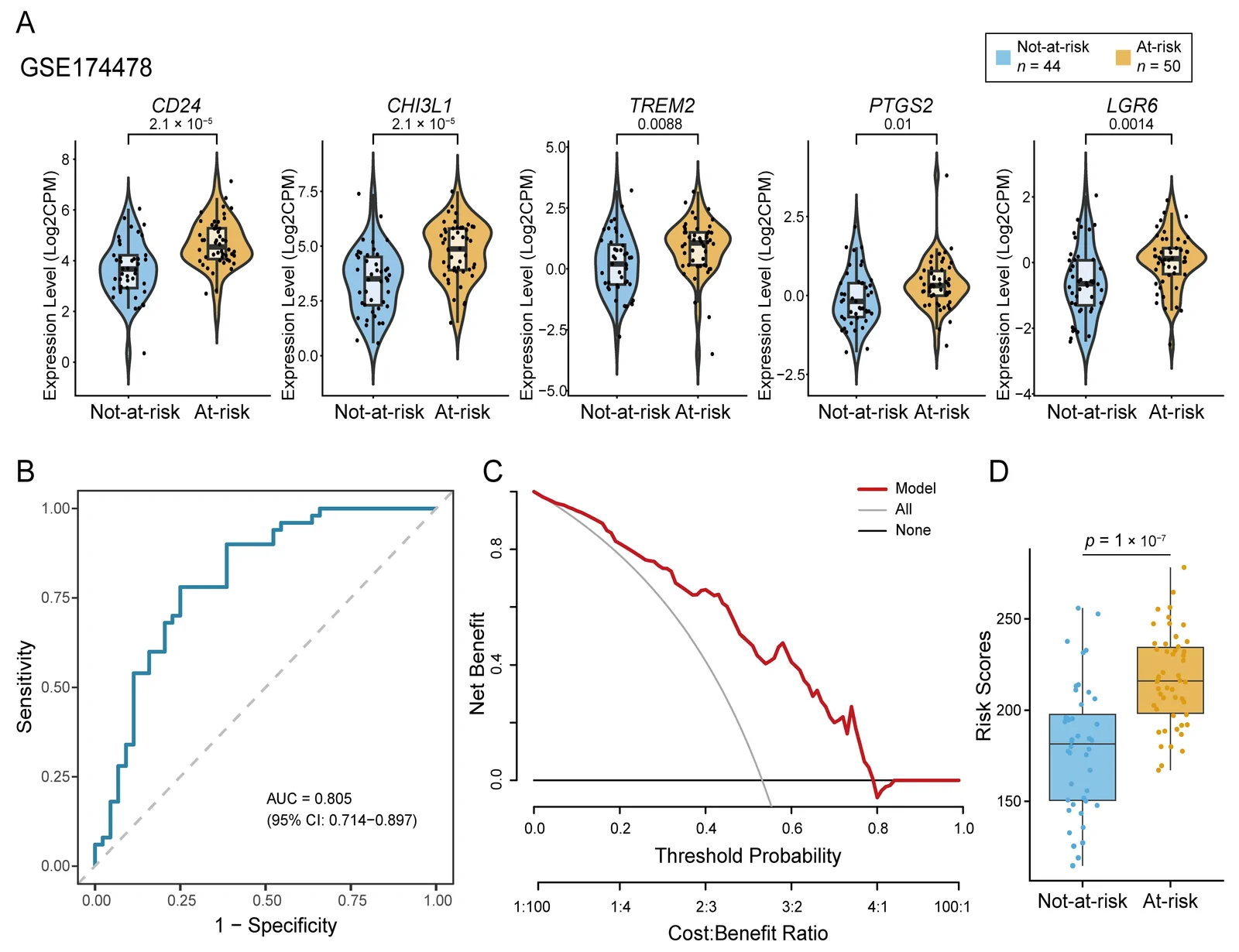
External validation of the nomogram model in GSE174478: (**A**) Violin plots showing expression levels of selected hub genes in the GSE174478 validation cohort. (**B**) Receiver operating characteristic (ROC) curve evaluating the discriminatory performance of the nomogram model. (**C**) Decision curve analysis (DCA) showing the potential clinical net benefit of the model. (**D**) Distribution of nomogram-derived risk scores in the validation cohort. Student’s *t*-test was used for group comparisons. Data are presented as mean ± SD.

The performance of the nomogram was further evaluated in the independent GSE174478 validation cohort. All five hub genes remained significantly upregulated in the at-risk group (all *p* < 0.05; Figure 6A). The nomogram retained favorable discriminatory performance, achieving an AUC of 0.805 (95% CI: 0.714–0.897) (Figure 6B). DCA further suggested the potential clinical net benefit of the model (Figure 6C). Moreover, nomogram-derived risk scores were significantly higher in the at-risk group (*p* = 1 × 10^−7^; Figure 6D), supporting the consistent discriminatory performance of the model in an independent public cohort.

### 3.6. Exploratory Plasma Proteomic Assessment of Candidate Biomarkers

**Figure 7:**
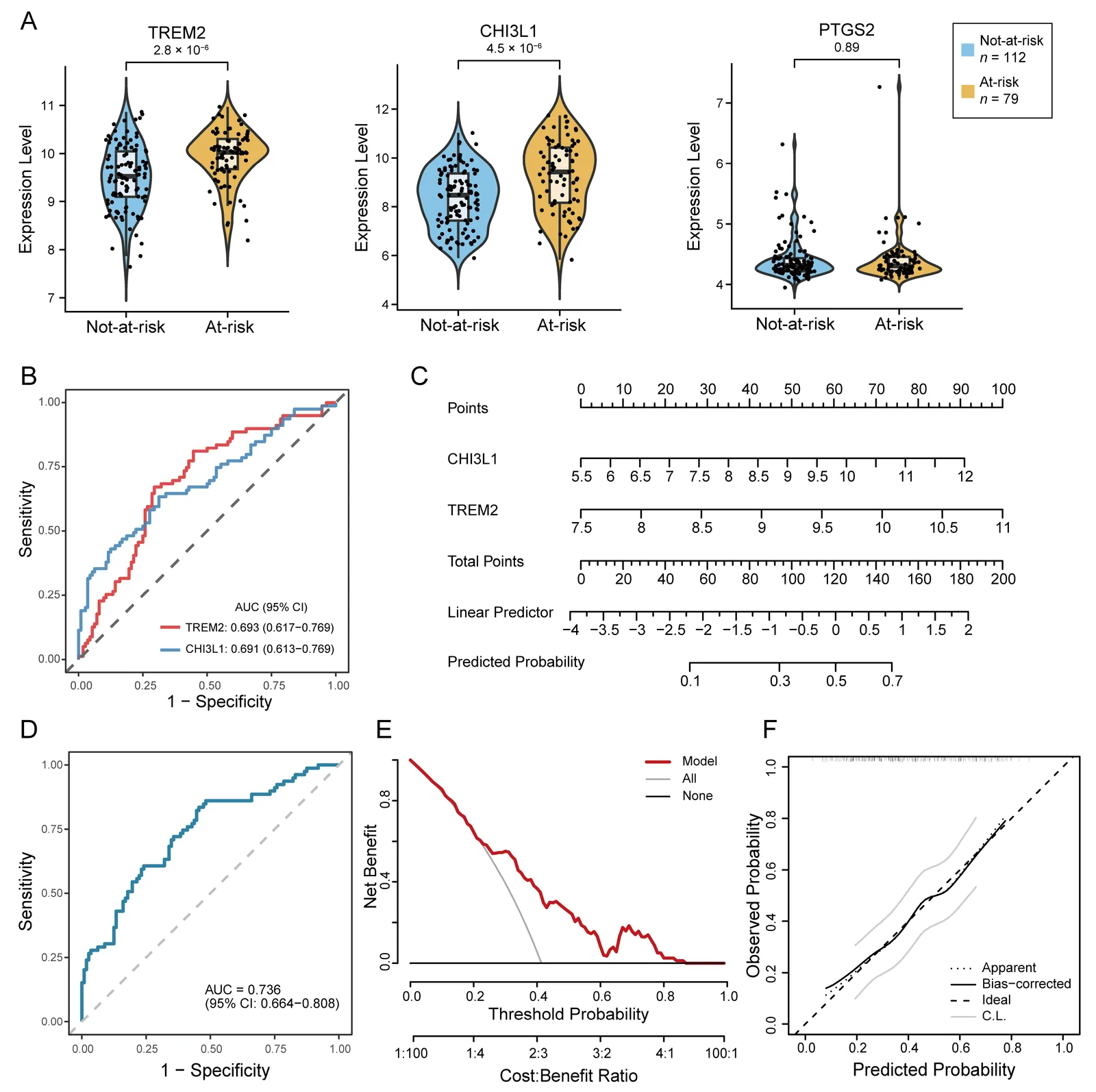
Exploratory plasma proteomic assessment of candidate biomarkers: (**A**) Violin plots showing plasma protein levels of TREM2, CHI3L1, and PTGS2 in the plasma proteomic cohort. (**B**) Receiver operating characteristic (ROC) curves for plasma TREM2 and CHI3L1. (**C**) Nomogram for calculating a risk score for at-risk MASH based on plasma TREM2 and CHI3L1 protein levels. (**D**) ROC curve evaluating the discriminatory performance of the plasma-based nomogram model. (**E**) Decision curve analysis (DCA) showing the potential clinical net benefit of the plasma-based model. (**F**) Calibration curve assessing agreement between predicted probabilities and observed outcomes of the plasma-based nomogram. Student’s *t*-test was used for group comparisons. Data are presented as mean ± SD.

To investigate whether tissue-derived candidate biomarkers could also be detected in circulation, plasma proteomic data from a previously published cohort were analyzed [17]. Plasma TREM2, CHI3L1, and PTGS2 were detected, whereas CD24 and LGR6 were not detected, which may be related to their predominantly membrane-associated and non-classically secreted nature [21,22]. TREM2 and CHI3L1 were significantly elevated in patients with at-risk MASH (both *p* < 0.0001), whereas PTGS2 showed no significant difference between groups (*p* = 0.89; Figure 7A). As individual biomarkers, plasma TREM2 and CHI3L1 demonstrated modest discriminatory performance, with AUC values of 0.693 (95% CI: 0.617–0.769) and 0.691 (95% CI: 0.613–0.769), respectively (Figure 7B).

A simplified nomogram integrating TREM2 and CHI3L1 achieved an AUC of 0.736 (95% CI: 0.664–0.808; Figure 7C,D), with acceptable calibration (Figure 7F) and potential net clinical benefit according to DCA (Figure 7E). The performance of this plasma-based model was numerically comparable to established non-invasive indices, including the Fibrosis-4 Index (FIB-4), NAFLD Fibrosis Score (NFS), and aspartate aminotransferase-to-platelet ratio index (APRI) (Table S7).

### 3.7. GSEA of Hub Genes

**Figure 8:**
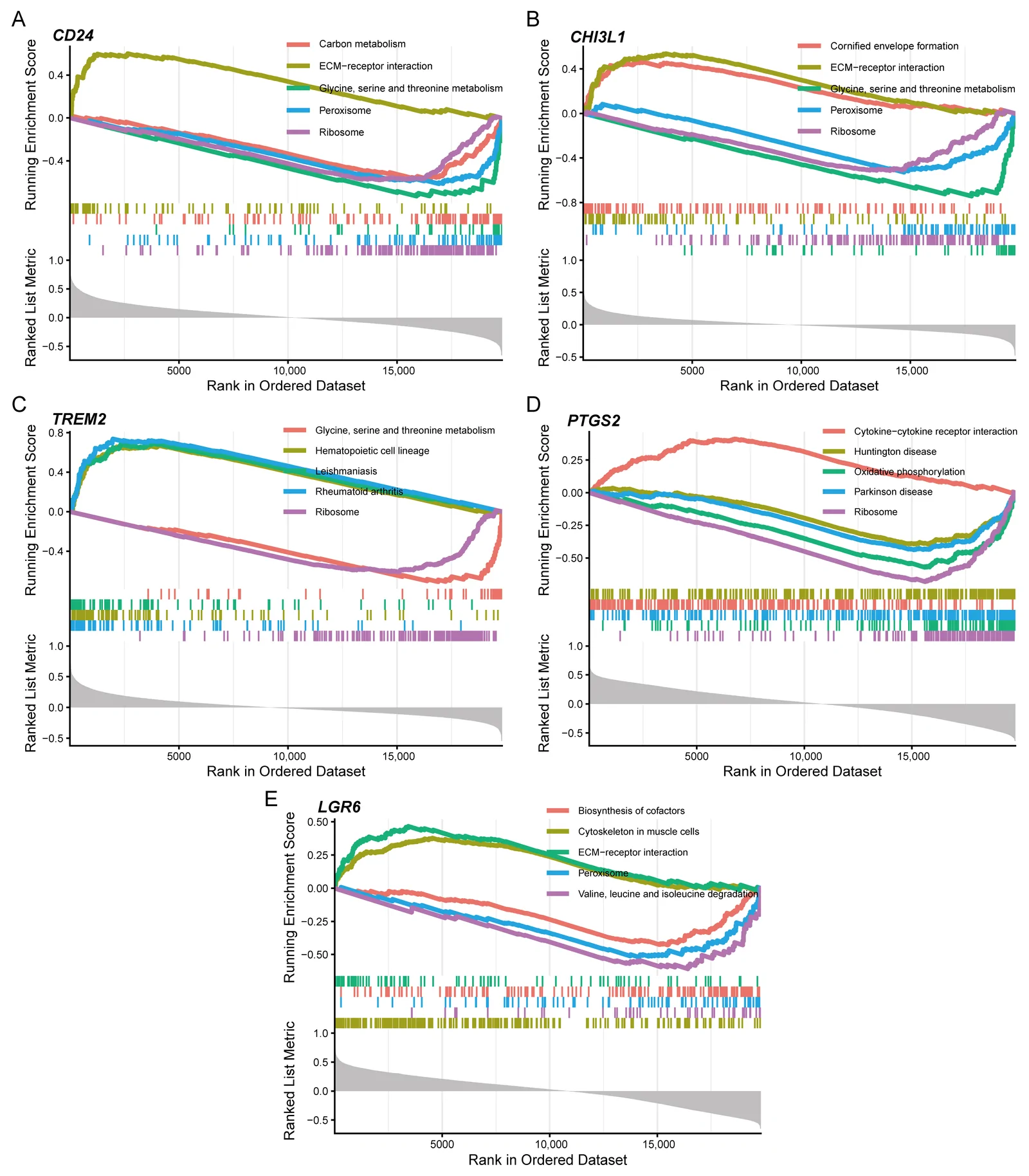
GSEA of hub genes: (**A**–**E**) Gene set enrichment analysis (GSEA) was performed for *CD24*, *CHI3L1*, *TREM2*, *PTGS2*, and *LGR6* based on ranked gene expression profiles. The top five enriched pathways associated with each gene are shown.

GSEA of the individual hub genes demonstrated distinct pathway-level enrichment patterns (Figure 8). High expression of *CD24*, *CHI3L1*, and *LGR6* was consistently associated with enrichment of extracellular matrix-receptor interaction pathways and metabolic processes, including amino acid metabolism and peroxisome-related pathways, whereas low expression was associated with ribosomal and carbon metabolism pathways (Figure 8A,B,E).

High *TREM2* expression was associated with enrichment of hematopoietic cell lineage and immune signaling pathways (Figure 8C). In contrast, high *PTGS2* expression was associated with cytokine–cytokine receptor interaction pathways, whereas low *PTGS2* expression was linked to oxidative phosphorylation and ribosomal pathways (Figure 8D).

### 3.8. Immune Infiltration Analysis and scRNA-Seq Expression Mapping

**Figure 9:**
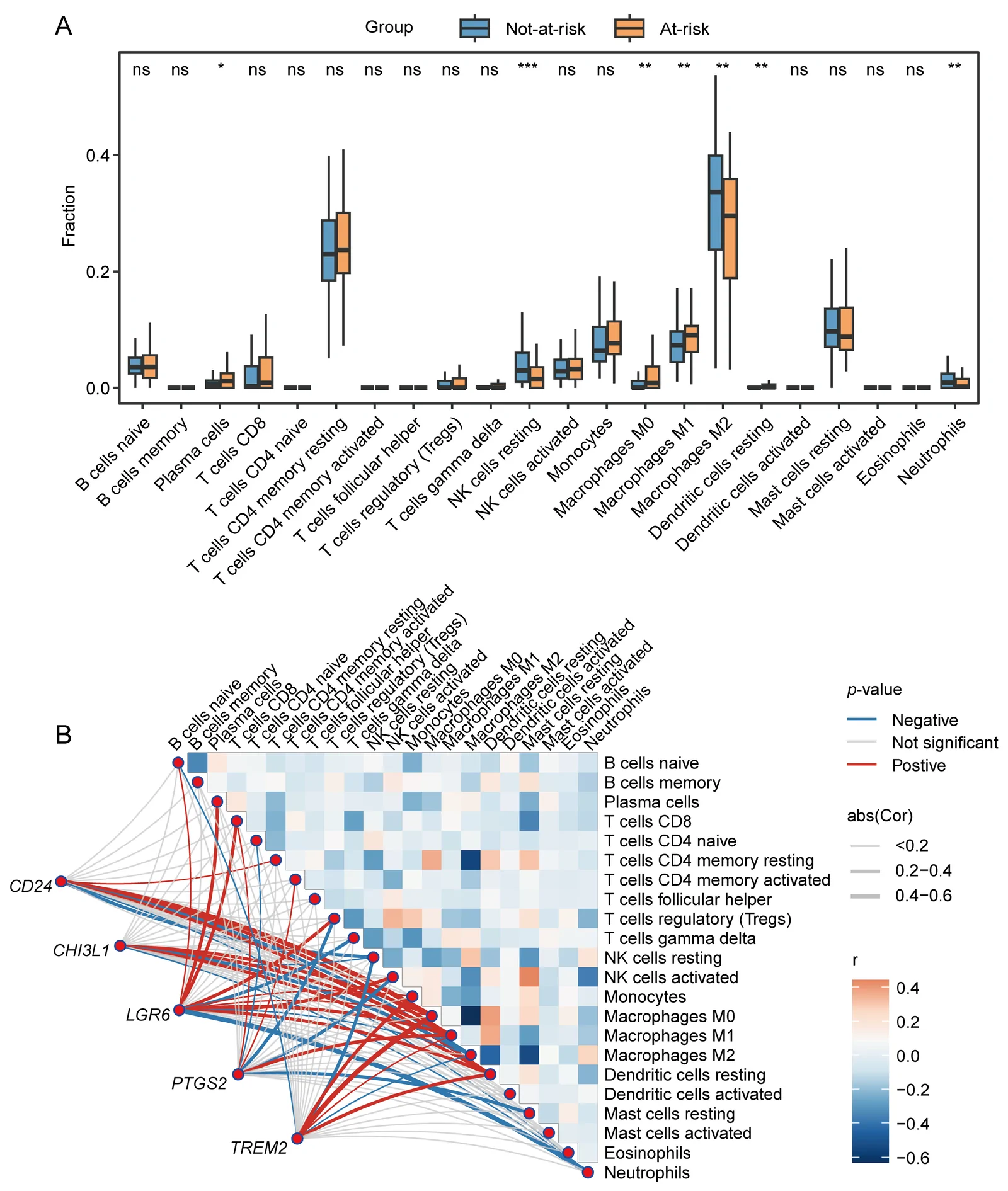
Immune infiltration analysis: (**A**) Boxplots showing the inferred proportions of immune cell subsets in the not-at-risk and at-risk groups. (**B**) Correlation heatmap showing associations between hub gene expression and inferred immune cell proportions. The Wilcoxon rank-sum test was used. Data are presented as medians with interquartile ranges. ns, not significant; * *p* < 0.05; ** *p* < 0.01; *** *p* < 0.001.

Immune cell composition was estimated using the CIBERSORT algorithm. Wilcoxon rank-sum testing showed that seven of the 22 immune cell subsets differed significantly between the at-risk and not-at-risk groups (Figure 9A). The inferred proportions of plasma cells, M0 and M1 macrophages, and resting dendritic cells were higher in at-risk MASH, whereas those of resting natural killer (NK) cells, M2 macrophages, and neutrophils were lower. Several hub genes also showed correlations with macrophage subsets (Figure 9B).

**Figure 10:**
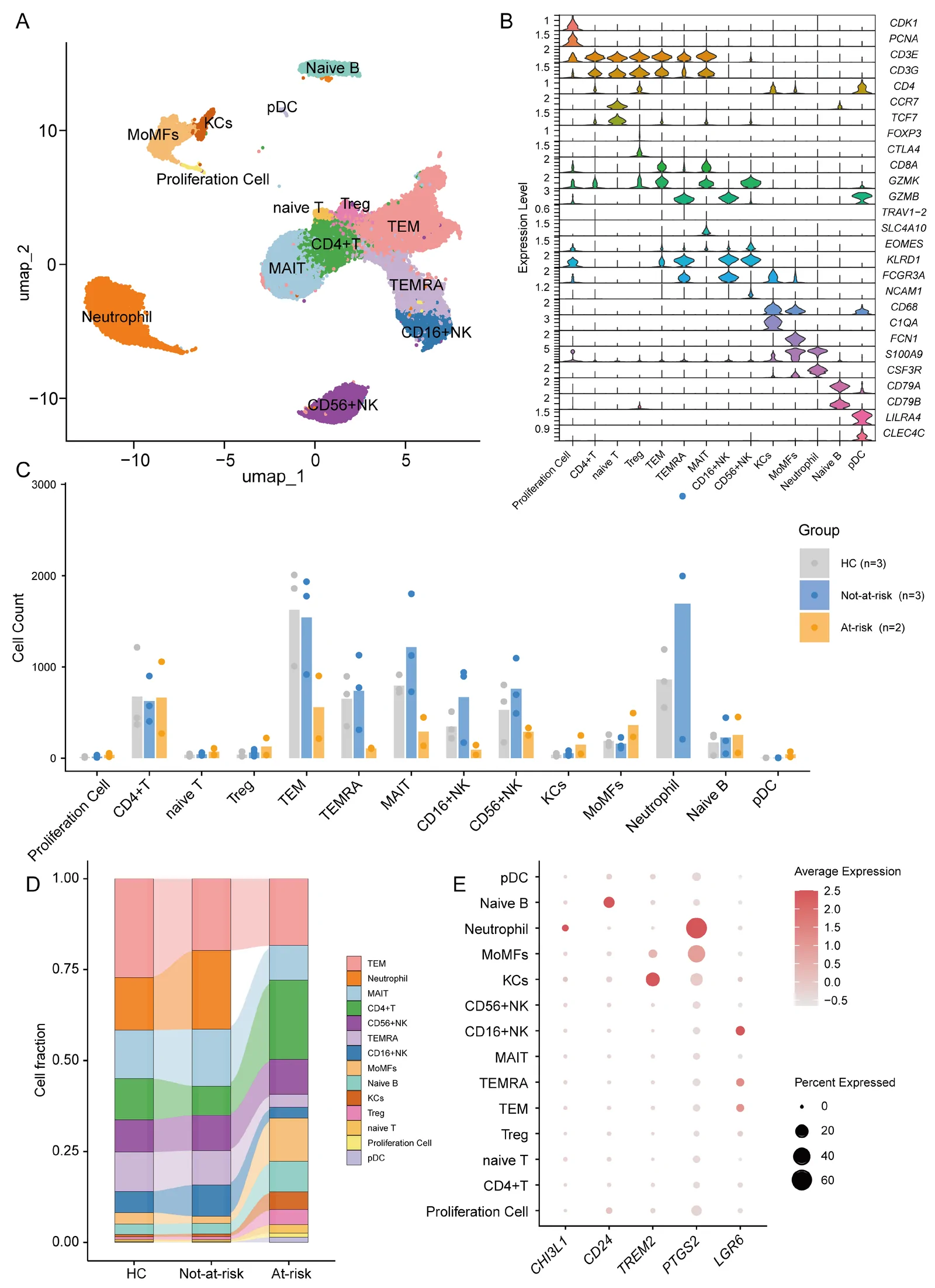
scRNA-seq analysis: (**A**) Uniform manifold approximation and projection (UMAP) plot of major immune cell populations from liver tissue. Colors are used to distinguish the annotated immune cell types. (**B**) Violin plots showing marker genes in different annotated cell types. Colors are used to distinguish the marker genes shown. (**C**) Cell counts of each immune cell population across the HC, not-at-risk, and at-risk groups. (**D**) Sankey diagram showing the relative proportions of immune cell populations. (**E**) Dot plot showing the average expression levels of hub genes (*CHI3L1*, *CD24*, *TREM2*, *PTGS2*, and *LGR6*) in different cell types. Abbreviations: TEM, effector memory T cells; TEMRA, terminally differentiated effector memory T cells re-expressing CD45RA; MAIT, mucosal-associated invariant T cells; Treg, regulatory T cells; MoMFs, monocyte-derived macrophages; KCs, Kupffer cells; pDC, plasmacytoid dendritic cells; NK, natural killer cells; HC, healthy controls.

These findings were further examined at the single-cell level using integrated liver scRNA-seq datasets comprising samples from healthy controls, patients with MASLD, and patients with MASLD cirrhosis (Figure 10A,B). At-risk samples consistently exhibited increased proportions of naïve B cells, Kupffer cells, and monocyte-derived macrophages, together with reduced proportions of CD16^+^ NK cells and neutrophils (Figure 10C,D).

Cell-type-specific expression profiling demonstrated that *CHI3L1* and *PTGS2* were predominantly expressed in neutrophils, whereas *PTGS2* was additionally expressed in macrophages. *CD24*, *TREM2*, and *LGR6* were preferentially expressed in naïve B cells, Kupffer cells, and CD16^+^ NK cells, respectively (Figure 10E).

### 3.9. Exploratory Molecular Regulatory Network and Drug–Gene Interaction Analysis

**Figure 11:**
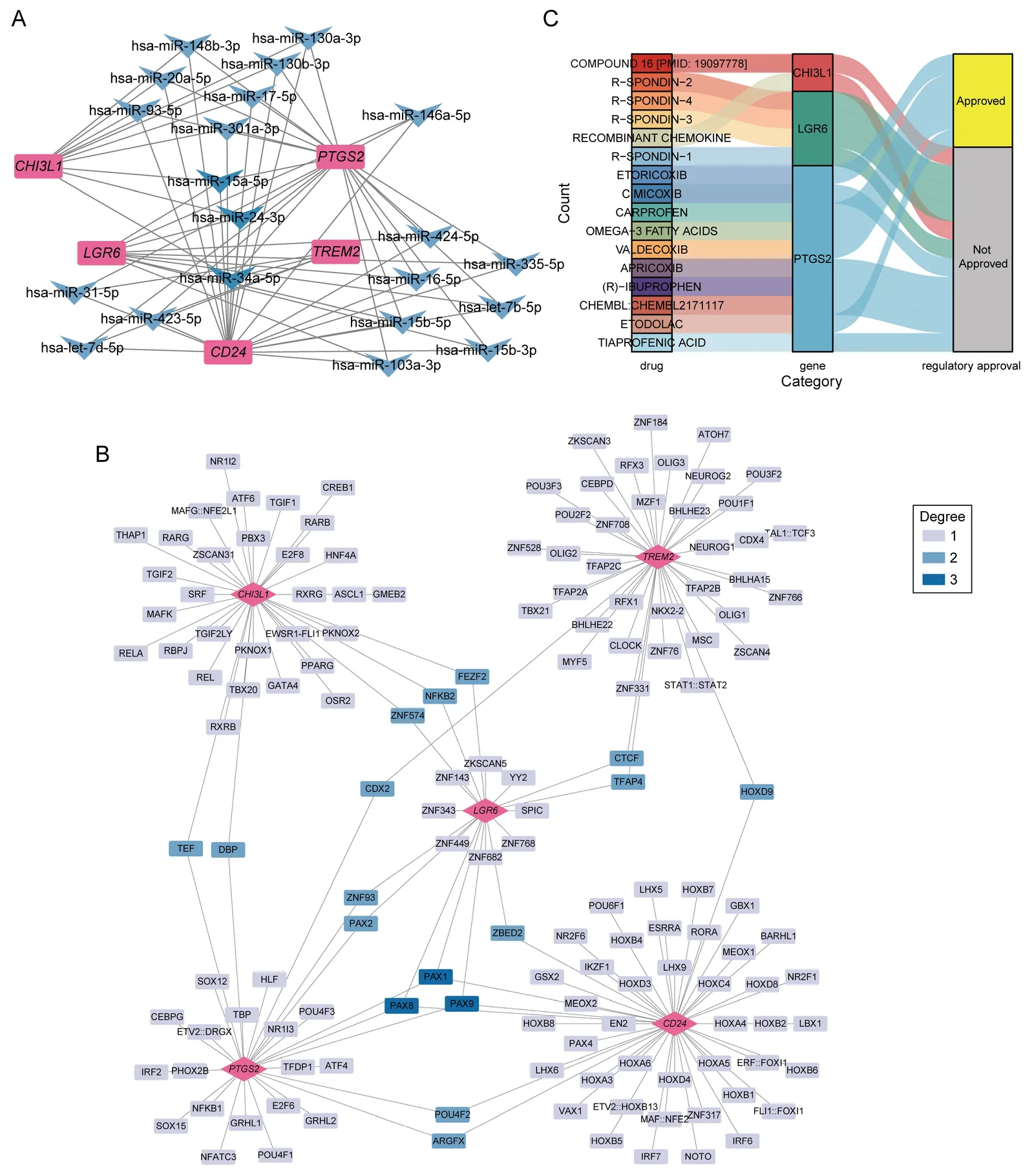
Molecular regulatory network and drug–gene interaction analysis: (**A**) Construction of the miRNA–hub gene network. (**B**) Construction of the transcription factor (TF)-hub gene network. (**C**) Exploratory drug–gene interaction analysis of the hub genes.

A total of 111 miRNAs associated with the hub genes were identified (Table S8), including 26 high-degree nodes forming a regulatory network comprising 66 interactions (Figure 11A). Transcription factor analysis identified 150 TFs predicted to regulate hub genes (Table S9), including paired box 1 (PAX1), PAX8, and PAX9 (Figure 11B).

An exploratory DGIdb query identified 16 reported drug–gene interactions involving *CHI3L1, PTGS2*, and *LGR6*, predominantly involving *PTGS2*, whereas no interactions were retrieved for *CD24* or *TREM2* (Figure 11C).

## 4. Discussion

In this exploratory study, we identified a five-gene efferocytosis-associated signature comprising *CD24*, *CHI3L1*, *TREM2*, *PTGS2*, and *LGR6* that distinguished histologically defined at-risk MASH from not-at-risk samples in public transcriptomic cohorts, achieving an AUC of 0.805 in the independent validation cohort. Additional murine expression validation and exploratory plasma proteomic analyses further supported the biological relevance of this candidate signature. Collectively, these findings suggest that efferocytosis-associated genes may serve as candidate biomarkers for identifying at-risk MASH.

Several NITs have been developed for risk stratification in MASLD. Most combine markers reflecting different biological aspects of disease, including hepatocellular injury, metabolic dysfunction, and imaging-derived features, as exemplified by the NIS4 [7], metabolomics-advanced steatohepatitis fibrosis score (MASEF) [23], and FibroScan-AST (FAST) score [24]. Compared with unrestricted transcriptome-wide screening, focusing on efferocytosis-associated genes narrows the candidate space to genes linked to a biologically relevant process implicated in MASH. Such a biologically informed signature may provide molecular information related to immune-mediated injury resolution and tissue remodeling, thereby offering a potentially complementary biological dimension to conventional biochemical or imaging-based indices [12]. If validated further, these biomarkers may warrant clinical evaluation as adjuncts to established NITs for identifying patients with at-risk MASH. Previously reported AUCs ranged from 0.534 to 0.700 for FIB-4, from 0.543 to 0.660 for NFS, and from 0.642 to 0.740 for APRI, whereas the five-gene nomogram achieved AUCs of 0.866 and 0.805 in the training and external validation cohorts, respectively (Table S7). However, prospective head-to-head validation is required to determine its comparative clinical utility.

The relevance of this candidate signature was further supported by murine expression validation and exploratory plasma analyses. In the animal model, the 24-week Western diet group exhibited more severe steatohepatitis-like injury and fibrotic remodeling than the 12-week group, partially recapitulating key histological features of at-risk MASH. Importantly, both mRNA and protein expression levels of all five hub genes were increased in the 24-week group, supporting their association with progressive diet-induced MASLD.

To explore whether this tissue-derived signature could also be reflected in circulating proteins, we analyzed a previously published proteomic cohort. Among the five hub genes, only TREM2 and CHI3L1 were detectable and differentially expressed in plasma, and a model based on these two proteins demonstrated moderate discriminatory performance, with an AUC of 0.736. CD24, PTGS2, and LGR6 were not incorporated into the plasma model, likely because tissue-level upregulation does not necessarily translate into detectable circulating protein signals. These proteins are predominantly membrane-associated or intracellular rather than classically secreted proteins, and their detection may additionally be influenced by protein stability, clearance kinetics, and proteomic platform coverage. The lower AUC of the plasma-based model relative to the transcriptomic model may also reflect greater interindividual variability in circulating protein concentrations and the influence of extrahepatic sources and systemic metabolic or inflammatory conditions, which may attenuate liver-specific molecular signals. In addition, the limited biological breadth of the two-marker panel may have constrained its discriminatory performance. Expanding the panel to include additional circulating markers related to efferocytosis and complementary inflammatory or fibrotic processes may improve discriminatory performance and should be evaluated in future studies. Commercial enzyme-linked immunosorbent assay kits are currently available for measuring soluble TREM2 and CHI3L1/YKL-40 in human serum or plasma, supporting the technical feasibility of targeted validation using conventional immunoassay methods. Nevertheless, clinical translation will require assay standardization, establishment of reference ranges and diagnostic cut-off values, prospective multicenter validation, and assessment of their incremental value beyond existing non-invasive tests. Overall, the plasma analysis provides preliminary evidence for the potential use of circulating biomarkers, while further clinical validation remains necessary.

Efferocytosis represents a central homeostatic mechanism linking hepatocyte death with either the resolution or persistence of inflammation and fibrotic remodeling in MASLD. Beyond preventing secondary necrosis, efferocytosis promotes pro-resolving macrophage reprogramming and limits chronic inflammatory signaling, thereby influencing the progression from steatohepatitis to fibrosis [25,26]. Recent mechanistic studies in MASH have demonstrated that impaired clearance of dying hepatocytes sustains hepatic inflammation and disrupts TREM2-dependent efferocytosis of lipid-laden apoptotic hepatocytes, resulting in their accumulation and persistent immune activation [15]. Furthermore, modulation of macrophage efferocytosis checkpoints has been shown to influence disease severity and fibrotic remodeling in metabolic steatohepatitis [14,27]. Collectively, these findings support efferocytosis as a biologically relevant process linking immune activation and fibrotic remodeling during MASH progression.

The five hub genes identified in this study converge on interconnected pathways involving immune regulation, metabolic stress, and tissue remodeling in MASLD. CD24 reflects context-dependent immune modulation at the interface between innate and adaptive immunity [28,29]. CHI3L1 integrates inflammatory activation with fibrogenic signaling through macrophage–stellate cell crosstalk [30]. TREM2 marks metabolically adapted macrophage subsets involved in apoptotic cell clearance and lipid stress responses [31,32]. Notably, comparison with previously reported efferocytosis-related signatures revealed partial cross-disease overlap, with *CD24* identified in a rheumatoid arthritis-associated signature and *CHI3L1* and *TREM2* included in an atherosclerosis-associated signature [33,34]. PTGS2 participates in prostaglandin-mediated inflammatory regulation with context-dependent effects on fibrosis and immune resolution [35,36], and efferocytosis-induced *PTGS2* expression has been linked to prostaglandin E2-dependent pro-resolving signaling [37]. LGR6, a receptor for the pro-resolving mediator maresin 1, promotes efferocytosis and may contribute to the restoration of hepatic homeostasis [38]; its role in enhancing macrophage efferocytosis has also been reported in intervertebral disc degeneration [39]. Together, these molecules may represent an efferocytosis-associated immunometabolic and tissue-remodeling program associated with at-risk MASH, while their partial overlap with efferocytosis-related signatures reported in other diseases suggests the presence of shared biological components across distinct inflammatory contexts.

The scRNA-seq analysis revealed distinct cell-type-specific expression patterns across the five hub genes. *TREM2* was preferentially expressed in Kupffer cells, consistent with its established association with macrophage-mediated recognition and clearance of apoptotic cells [15], and mIHC further confirmed its localization to CD68^+^ hepatic macrophages. By contrast, *CHI3L1* and *PTGS2* were enriched mainly in neutrophils, *CD24* in naïve B cells, and *LGR6* in CD16^+^ NK cells. Together, these findings provide cellular context for the signature and suggest that it captures a multicellular immune and tissue-remodeling program associated with efferocytosis-related disease processes. Further functional studies are needed to clarify the contribution of each gene to these processes.

This study has several limitations. First, the reliance on cross-sectional transcriptomic datasets precluded assessment of temporal gene expression dynamics and causal inference during MASLD progression. Although the identified signature was externally validated in an independent GEO cohort, validation remained confined to retrospective public datasets; therefore, its generalizability and potential value for predicting subsequent disease progression require further evaluation in prospective, independent, multicenter clinical cohorts. The ethnic and geographic composition of the GEO cohorts was also incompletely characterized, which may limit generalizability across populations. Second, incomplete clinical annotations within the public datasets, particularly regarding metabolic, biochemical, and demographic variables, limited further clinical stratification and adjustment for potential confounders, including age, sex, body mass index, diabetes status, liver enzyme levels, and metabolic profiles. This limitation also precluded a direct head-to-head comparison of the nomogram developed in this study with established clinical scoring systems. Third, the experimental validation was conducted using a relatively small number of animals, which may limit the generalizability of the findings, and did not include a baseline group, which precluded assessment of changes occurring during the first 12 weeks of Western diet feeding. Finally, the mechanistic and cell-type-specific roles of the five hub genes in MASLD remain incompletely understood. Future functional studies, including conditional gene deletion in relevant hepatic cell populations, are therefore required to clarify their causal contributions and therapeutic relevance.

## 5. Conclusions

In conclusion, this study identified five efferocytosis-associated hub genes, *CD24*, *CHI3L1*, *TREM2*, *PTGS2*, and *LGR6*, through integrated bioinformatic analyses and constructed a gene-based model that demonstrated moderate-to-good discriminatory performance in an independent public validation cohort. These findings support the potential relevance of efferocytosis-associated signatures for identifying histologically defined at-risk MASH. We also explored a simplified plasma-based model incorporating TREM2 and CHI3L1, which showed moderate discriminatory performance. Nevertheless, prospective clinical validation and mechanistic studies are required to clarify the generalizability, causal relevance, and cell-type-specific roles of these genes in MASLD progression.

## Data Availability

The datasets supporting the conclusions of this study are publicly available in the GEO database under accession numbers GSE135251, GSE174478, GSE186328, and GSE136103. Plasma proteomics data were obtained from a previously published study via https://doi.org/10.1038/s42255-023-00775-1. All code used for the analyses is available from the corresponding author upon reasonable request.

## Supplementary Materials

The following supporting information can be downloaded at https://www.mdpi.com/article/10.3390/genes17080948/s1, Table S1: Summary of datasets used in this study; Table S2: Efferocytosis-related genes; Table S3: Real-time polymerase chain reaction primers; Table S4: Antibodies used in this study; Table S5: Differentially expressed genes; Table S6: Risk-associated efferocytosis-related genes; Table S7: Reported diagnostic performance of commonly used non-invasive tests for MASLD; Table S8: Predicted miRNAs targeting hub genes; Table S9: Predicted transcription factors targeting hub genes.

## Abbreviations

The following abbreviations are used in this manuscript:

MASH	Metabolic dysfunction-associated steatohepatitis
NAS	Nonalcoholic fatty liver disease activity score
NITs	Non-invasive tests
MASLD	Metabolic dysfunction-associated steatotic liver disease
ERGs	Efferocytosis-related genes
GEO	Gene Expression Omnibus
scRNA-seq	Single-cell RNA sequencing
KEGG	Kyoto Encyclopedia of Genes and Genomes
FDR	False discovery rate
log2FC	log2 fold change
DEGs	Differentially expressed genes
ssGSEA	Single-sample gene set enrichment analysis
GO	Gene Ontology
GSEA	Gene set enrichment analysis
ALT	Alanine aminotransferase
AST	Aspartate aminotransferase
LASSO	Least absolute shrinkage and selection operator
SVM-RFE	Support vector machine-recursive feature elimination
ORs	Odds ratios
CIs	Confidence intervals
ROC	Receiver operating characteristic
AUC	Area under the curve
DCA	Decision curve analysis
RT-qPCR	Reverse transcription-quantitative polymerase chain reaction
H&E	Hematoxylin and eosin
mIHC	Multiplex immunohistochemistry
CD	Cluster of differentiation
CIBERSORT	Cell-type identification by estimating relative subsets of RNA transcripts
TFs	Transcription factors
DGIdb	Drug Gene Interaction Database
FIB-4	Fibrosis-4 index
NFS	NAFLD fibrosis score
APRI	Aspartate aminotransferase-to-platelet ratio index
SD	standard deviation
SEM	standard error of the mean
IQR	interquartile range
UMAP	Uniform manifold approximation and projection
IL	Interleukin
NK	Natural killer
TEM	Effector memory T cells
TEMRA	Terminally differentiated effector memory T cells re-expressing CD45RA
MAIT	Mucosal-associated invariant T cells
Treg	Regulatory T cells
MoMFs	Monocyte-derived macrophages
KCs	Kupffer cells
pDCs	Plasmacytoid dendritic cells
HC	Healthy controls
MASEF	Metabolomics-advanced steatohepatitis fibrosis score
FAST	FibroScan-AST
PAX	Paired box
